# Minoxidil as a treatment for onychodystrophy: a systematic review

**DOI:** 10.1016/j.jdin.2025.05.014

**Published:** 2025-06-23

**Authors:** Anagha B. Thiagarajan, Jack P. Woll, Milan M. Hirpara, Natasha A. Mesinkovska, Luke Horton

**Affiliations:** Department of Dermatology, University of California Irvine School of Medicine, Irvine

**Keywords:** epidemiology, minoxidil, nail dystrophy, onychodystrophy

*To the Editor:* Onychodystrophy, defined by abnormal changes in the shape, color, texture, or growth of nails, can arise secondary to numerous etiologies, with treatment typically targeting the underlying cause.^1^ However, some cases are idiopathic, where nail-targeted therapy could be of great clinical utility.^2^ Recently, minoxidil has shown efficacy in treating onychodystrophy.^1^^,^^3^ This systematic review evaluates the existing literature on minoxidil as a treatment for onychodystrophy.

This review followed the Preferred Reporting Items for Systematic Reviews and Meta-Analyses (PRISMA) 2020 guidelines and was registered on the International Prospective Register of Systematic Reviews (PROSPERO) website (CRD42024622702). The PubMed, Web of Science, and Scopus databases were searched with the following strategy: “Minoxidil AND nail.” Studies investigating minoxidil use for the treatment of nail dystrophy published between January 1, 2000, and December 1, 2024, were included. Background articles, review articles, studies with nonhuman subjects, and studies in languages other than English were excluded.

Among 108 references identified, 6 were included, yielding 197 patients (61 male, 136 female) (Fig 1). Five out of 6 studies evaluated nail growth as an endpoint for minoxidil treatment (*n* = 196), while 1 study each assessed nail appearance (*n* = 66), nail color (*n* = 1), and nail strength (*n* = 66) (Table I). 3 out of 6 studies used 5% topical minoxidil (*n* = 120), 1 used 2% topical minoxidil (*n* = 1), and 1 used 1.25 mg or 2.5 mg oral minoxidil daily (*n* = 76).

**Fig 1:**
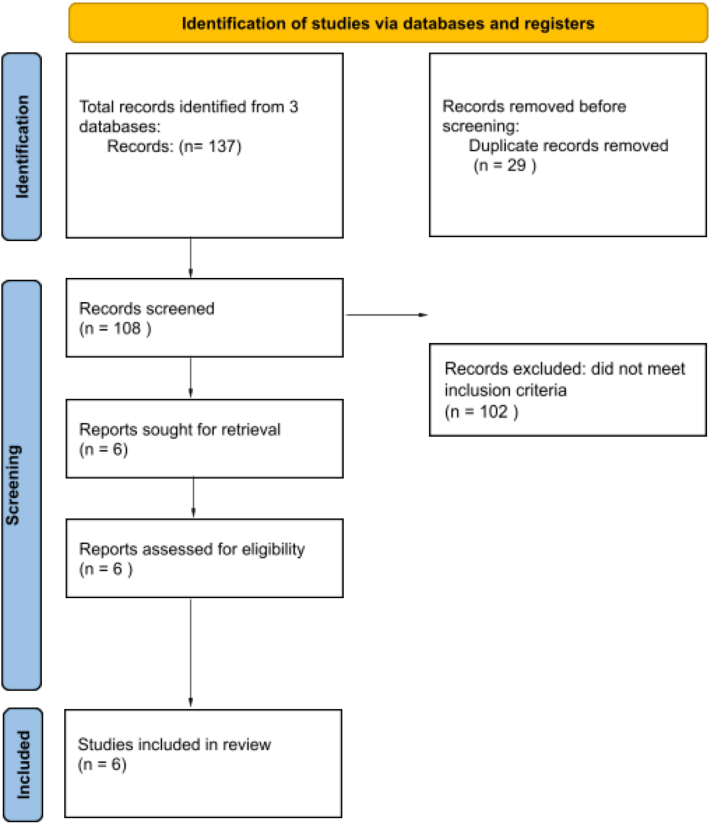
PRISMA flow diagram for identification, screening, inclusion, and exclusion of studies in this systematic review. *PRISMA,* Preferred Reporting Items for Systematic Reviews and Meta-Analyses.

**Table I:** Summary of included studies evaluating minoxidil for onychodystrophy

Author (y)	Treatment regimen (symptoms treated)	Nails treated (dosing)	Study type, sample size (% female)	Metric evaluated	Results
Kumpol A. (2017)^1^	5% topical minoxidil (growth)	2 nails per hand per subject (twice daily)	Pilot study, N = 32 (50%)	Change in nail growth rate from baseline (mm)	**Week 4:** 3.91 (control) vs 4.27 (treatment), *P* = .001 **Week 8:** 6.93 (control) vs 7.46 (treatment), *P* <.001
Algain M. (2021)^2^	2% topical minoxidil (discoloration)	4 nails (2 fingernails and 2 toenails) (daily)	Case report, N = 1 (100%)	Presence of discoloration	**Month 6:** discoloration resolved on all treated nails, excluding the distal half of both big toes
Alsalhi W. (2023)^3^	Oral minoxidil 1.25 mg (growth, strength, appearance)	All fingernails and toenails (daily)	Longitudinal survey study, N = 66 (71%)	Self-evaluation of nail growth, strength, and appearance	**Month 6:** 36.4% reported nicer-looking nails **Month 7:** 53% reported increased growth **Month 8:** 37.9% reported stronger nails
Starace M. (2023)^4^	5% topical minoxidil, 3 400 IU vitamin E tablets (growth,onychomadesis)	Symptomatic toenails treated (as needed once daily), and vitamin E (daily)	Retrospective survey study, N = 50 (92%)	Self-evaluation of treatment response (scale of 0-3, where 0 = no response, 3 = optimal response)	*Growth responses:***Month 6:** zero (7%), 1 (59%), 2 (26%), 3 (8%) **Month 12:** zero (1%), 1 (18%), 2 (46%), 3 (35%)
Garbers L. (2021)^5^	5% topical minoxidil (growth)	2 nails per hand per subject (daily)	Experimental study N = 38 (68%)	Nail growth rate relative to: (1) control, oral 2.5 mg. (2) biotin, oral 2.5 mg (3) biotin + topical minoxidil (2.5 mg, 5%)	*Results of minoxidil relative to control*: **Day 14:** higher than control (*P* <.01)∗**Day 28:** 19% increase in growth rate (*P* <.01)
Barbosa B. (2024)	Oral minoxidil 1 mg, 2.5 mg (growth)	Thumb nails per hand per subject (daily)	Randomized control trial N = 10 (0%)	Change in nail growth from the thumbnail lunula and markings on the nail plate	*Results of oral minoxidil 1 mg relative to control:***Day 14:** no significant increase in growth relative to control *Results of oral minoxidil 2.5 mg relative to control:***Day 14:** increase in nail growth speed by 50.7% (*P* <.01)

∗ *P* value listed, but no growth rate provided.

Topical minoxidil (5%) demonstrated increases in nail growth as early as 14 days, with 1 study finding a 19% increase in nail growth rate relative to control at 28 days (*P* < .0001).^5^ In another study, topical minoxidil (5%) was found to increase nail growth rate relative to untreated nails on the same hand (6.93 mm vs 7.46 mm; *P* < .001) evaluated at 8 weeks.^1^ In a case of yellow nail syndrome, topical minoxidil (2%) treatment resulted in the resolution of fingernail and toenail discoloration within 6 months of starting treatment, with no recurrence at 72 months.

Oral minoxidil also showed promise, with 1 study showing that oral minoxidil (1.25 mg) improved nail appearance in 36.4% of patients at month 6, nail growth in 53.0% of patients at month 7, and nail strength in 37.9% of patients at month 8.^3^

This systematic review demonstrates initial promising data for minoxidil as a treatment for onychodystrophy with improvement in color, texture, strength, and growth rate. The beneficial effect of minoxidil on onychodystrophy is hypothesized to be related to increased endothelial growth factors, leading to improved blood perfusion and nail growth.^3^^,^^4^

While all 6 included studies (*n* = 197) demonstrated statistically significant improvement in the measured onychodystrophy outcomes, the low number of included studies (*n* = 6) and the limited use of blinding and controls in these studies limits the capacity for broader inference. Lack of standardization in onychodystrophy diagnosis, short-term study length, and heterogeneity of treatment regimens and measured outcomes further contribute to limited generalizability. It should also be noted that reporting bias for favorable results may positively skew the outcomes presented in the literature and reflected in this study.

## Conflicts of interest

None disclosed.
